# Effect comparison of three different types of transanal drainage tubes after anterior resection for rectal cancer

**DOI:** 10.1186/s12893-020-00811-x

**Published:** 2020-07-25

**Authors:** Yun Luo, Chang-Kang Zhu, Ding-Quan Wu, Liang-Bi Zhou, Chong-Shu Wang

**Affiliations:** 1 Department of General Surgery, Beibei Traditional Chinese Medical Hospital, Chongqing, China; 2 Department of Surgery, Colorectal Cancer Center, Nanchong Oriental Hospital, Nanchong, Sichuan Province China

**Keywords:** Transanal drainage tube, Anastomotic leakage, Rectal cancer

## Abstract

**Background:**

Anastomotic leakage (AL) is one of the most severe early complications after rectal cancer surgery. Many studies and meta-analysis results show that the indentation of transanal drainage tubes (TDT) can prevent and reduce the incidence of AL. However, the size and material of drainage tubes are rarely reported. Herein, we compare the effect of three kinds of TDT and analyze the use of TDT material and size to prevent AL, which may better prevent the occurrence of AL.

**Methods:**

The clinical data of 182 patients who underwent laparoscopic anterior resection of rectal cancer were retrospectively analyzed between January 2016 and March 2019. According to the types of indwelling TDT after the operation, they were divided into Fr32 silicone tubes (81 cases), Fr24 silicone tubes (54 cases), Fr24 latex tubes (47 cases). The first drainage, exhaust, defecation, abdominal distension and anastomotic leakage of the patients with three different types of TDT were compared.

**Results:**

There was no significant difference in the degree of first exhaust, abdominal distension and anastomotic leakage among three different types of TDT; the time of first drainage and defecation of the Fr32 silicone tube was significantly earlier than that of Fr24 silicone tube and Fr24 latex tube.

**Conclusion:**

The drainage effect of the Fr32 silicone tube is better than that of Fr24 silicone tube and Fr24 latex tube after anterior resection for rectal cancer, Fr32 silicone may better prevent the occurrence of AL, but randomized controlled studies are needed.

## Background

Rectal cancer is one of the most common malignant tumors in China, accounting for 60–70% of colorectal cancer [1]. Moreover, the low rectal cancer accounts for 60–75% of rectal cancer [2]. With the promotion of the concept of total mesorectal excision (TME), the development of laparoscopic surgery techniques, and the application of related anastomotic instruments, the anus-preserving rate of patients with low and medium rectal cancer continues to increase. However, AL is one of the most severe early complications after the operation, with an incidence of 1.3% ~ 23.0% reported in the literature [3–5]. Proximal colon or ileum stoma is the most commonly used method of prevention and treatment, but it requires secondary surgeries and produces many complications, which are controversial [6].

In 1997, Klein et al. proposed that TDT is an effective method to prevent the AL [7]. Many prospective studies and meta-analysis results show that the indentation of TDT can prevent and reduce the incidence of anastomotic leakage [8–13]. Transanal tube placement to prevent AL has become a routine choice for surgeons in recent years, but there is no uniform type of TDT in clinical practice. In this study, 182 cases of anterior resection (AR) of rectal cancer were studied in our hospital from January 2016 to March 2019. In patients with Dixon operation, we analyzed the use of TDT material and size to prevent AL, and the summary report is as follows.

## Methods

### Patients and TDT

**Fig. 1:**
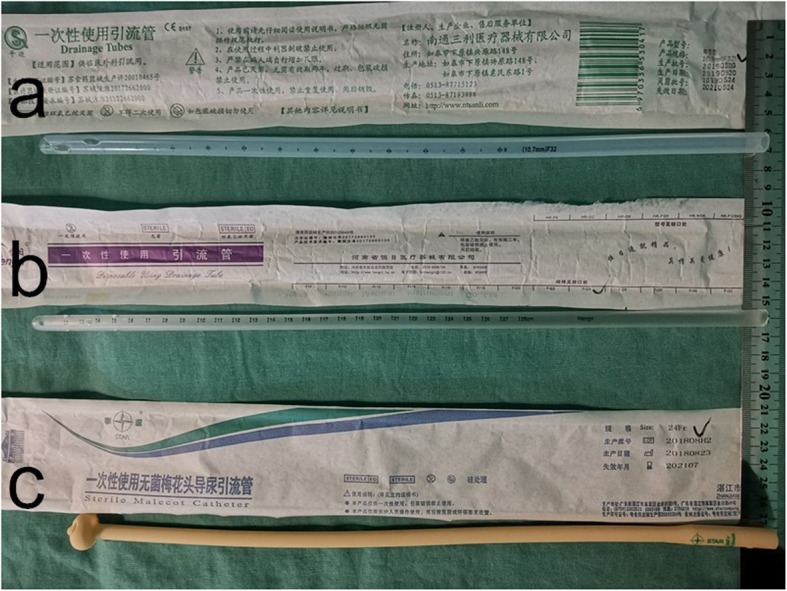
Three different types of TDT, **a**: Fr32 silicone tube, **b**: Fr24 silicone tube, **c**: Fr24 latex tube

**Fig. 2:**
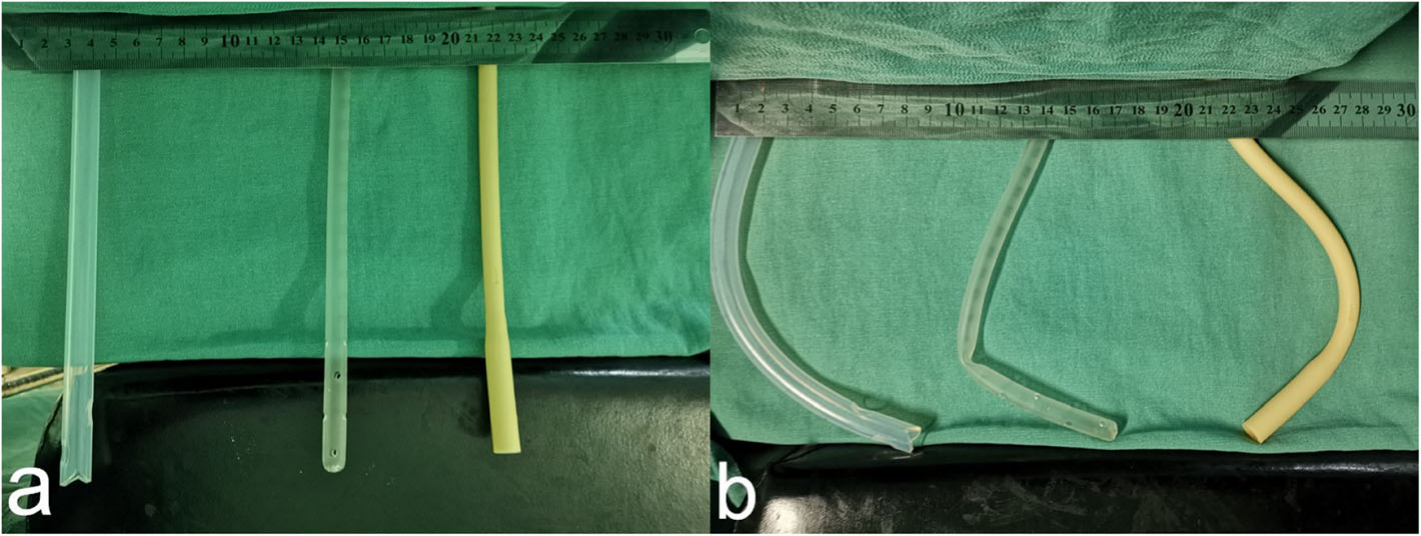
Diameter, hardness and flexibility of three different types of TDT, **a**: in the natural state, **b**: at the same force.

Retrospective data were collected from 182 patients who underwent anterior resection laparoscopic rectal cancer surgery between January 2016 and March 2019(approved by our hospital ethics committee), including 85 males and 97 females, aged 47–81 (62 ± 16.8) years. Among the indwelling TDT, there were 81 cases of Fr32 silicone tube, with a diameter of about 10.0 mm and a hard texture, which was not easy to fold, small or close, and mainly used for closed thoracic drainage tube. In 54 cases, the inner diameter of the Fr24 silicone tube was about 7.6 mm, which was soft and easy to fold, shrink or close, mainly used for abdominal drainage tube. Fr24 latex tubes were used in 47 cases, which of the internal diameter was about 7.6 mm, and it was flexible and relatively challenging to fold, shrink or close, mainly used for catheters or T tubes. Three different types of TDT was showed in Fig. 1 and Fig. 2a, b. The inner end of TDT was cut into “V”, and the proximal end was cut into 3–4 side holes in different directions.

### Case inclusion criteria

Inclusion criteria were as follows:(1) all patients were diagnosed as rectal cancer by colonoscopic biopsy before the operation, and CT or MRI found no distant metastasis; (2) standard total mesorectal excision anus-preserving operation was performed by the director of gastrointestinal surgery, the anastomosis tool used was American Johnson & Johnson Ethicon Intraluminal Circular Stapler, and all patients did not have preventive stoma; (3) TDT were placed on anastomotic stoma and detained for 5–7 days after the operation; (4) the operation time was between 2 and 4 h, and the duration of anesthesia was between 2.5 and 4.5 h; (5) none of these patients received preoperative chemotherapy or radiation therapy.

### Case exclusion criteria

The criteria for excluding patients included: (1) diabetes mellitus, cardiovascular and cerebrovascular diseases, liver, kidney, lung and other primary diseases; (2) mental disorders; (3) re-abdominal surgery with severe intestinal adhesion; (4) preoperative anemia, low protein and severe malnutrition; (5) intraoperative bleeding of more than 400 ml, or intraoperative or postoperative blood transfusion; (6) Severe complications, infections, deterioration of the disease occurred within 12 h after the operation, or secondary surgery was needed for various reasons; (7) Postoperative analgesia lasted more than 72 h.

### Definition of AL

The occurrence of AL should be considered as follows [14, 15, 1] persistent fever with unknown infection signs (no other infection focus); (2) turbid liquid or fecal or infectious pus, sometimes mixed with gas, was found in the drainage fluid of abdominal cavity or presacral drainage tube; (3) patients had signs of peritonitis, and digital rectal examination can touch the rectal anastomosis; (4) pelvic effusion or abscess or free gas can be found by ultrasound or CT examination, and contrast media can leak out of the leak or drain out of the abdominal drainage tube by digestive tract angiography.

### Judgment of the first postoperative drainage, exhaust and defecation

Postoperative first drainage time: the time when the patient first drained liquid from TDT or anus after the operation, including intestinal fluid, exudate, blood and other liquids.

Postoperative first exhaust time: the patients themselves mainly determined the time of postoperative first exhaust..

Postoperative first defecation time: feces are seen in the TDT or excrement is discharged from the anus.

### Criteria for abdominal distension

Patients with Postoperative abdominal distension score standard [16]: Grade 0 (0 score): no feeling of abdominal distension; Grade 1 (1 score): mild abdominal distension, slightly higher abdominal wall tension, does not affect rest and sleep; Grade 2 (2 scores): moderate abdominal distension, abdominal wall tension, affecting rest and sleep; Grade 3 (3 scores): severe abdominal distension, abdominal wall tension, cannot rest and sleep.

### Statistical analysis

SPSS 21.0 statistical software was used for data analysis. The measurement data were analyzed by variance analysis and expressed in the form of mean + standard deviation. The counting data were compared by the chi-square test or Fisher’s exact probability method for clinical characteristics in three different types of TDT. A value of *P* < 0.05 was considered statistically significant.

## Results

### Comparison of patient characteristics

There was no statistically significant difference in the general clinical data of patients with gender, age, tumor stage and complicated chronic diseases among the three different types of TDT. There was good comparability between the three groups, and those data are shown in Table 1.

**Table 1:** clinical characteristics of patients [case (%)]

Characteristics	Fr32 silicone tube(*n* = 81)	Fr24 silicone tube(*n* = 54)	Fr24 latex tubes(*n* = 47)	*χ*^***2***^ value	*P* value
Gender				0.284	0.868
Male	45 (55.56)	30 (55.56)	24 (51.06)		
Female	36 (44.44)	24 (44.44)	23 (48.94)		
Age (years)				3.916	0.141
<60	30 (37.04)	15 (38.46)	25 (53.19)		
≥60	51 (62.96)	39 (61.54)	22 (46.81)		
Hemoglobin(g/ L)				0.513	0.774
≤90	12 (14.81)	8 (14.81)	5 (10.64)		
> 90	69 (85.19)	46 (85.19)	42 (89.36)		
Albumin (g/ L)				0.369	0.820
≤30	8 (9.88)	7 (12.96)	6 (12.77)		
> 30	73 (90.12)	47 (87.04)	41 (87.23)		
Chronic diseases (diabetes, etc.)				3.328	0.189
Yes	35 (43.21)	15 (38.46)	17 (36.17)		
No	46 (56.79)	39 (61.54)	30 (63.83)		
Tumor diameter (cm)				1.190	0.385
≤ 3	28 (34.57)	14 (25.93)	18 (38.30)		
>3	53 (65.43)	40 (74.07)	29 (61.70)		
Anastomotic level (cm)				1.373	0.503
≤ 5	19 (23.46)	16 (29.63)	10 (21.28)		
>5	62 (76.54)	36 (70.37)	37 (78.72)		
Tumor stage				0.362	0.849
T1–2	17 (20.99)	10 (18.52)	8 (17.02)		
T3–4	64 (79.01)	44 (81.48)	39 (82.98)		
Regional lymph nodes				1.863	0.394
N0–1	58 (71.60)	43 (79.63)	32 (68.09)		
N2	23 (28.40)	11 (20.37)	15 (31.91)		

### Comparison of research indicators

There was no significant difference in the time of first exhaust and the scores of postoperative abdominal distensions among three different types of TDT. However, there was a significant difference in the time of the first drainage and defecation. The time of the first drainage and defecation of the Fr32 silicone tube was significantly earlier than that of the Fr24 silicone tube and the Fr24 latex tube, and it is shown in Table 2. The incidence of AL among the study subjects after rectal cancer surgery was 3.85%. Due to the small number of cases of AL, Fisher’s exact probability method was used for comparison, and there was no statistically significant difference in the incidence of AL among the three different types of TDT, as shown in Table 3. The number of leaks might be too small to permit formal regression analysis.

**Table 2:** comparison of drainage, exhaust, defecation and abdominal distention in three different types of TDT(^−^x ± s).

Variables	Fr32 silicone tube(*n* = 81)	Fr24 silicone tube(*n* = 54)	Fr24 latex tubes(*n* = 47)	*F* value	*P* value
First drainage time (hours)	3.35 ± 1.42	4.50 ± 1.85	4.34 ± 1.59	10.318	0.000
First exhaust time (days)	3.16 ± 0.98	3.37 ± 1.00	3.34 ± 1.20	0.798	0.452
First defecation time (days)	3.47 ± 0.79	4.13 ± 0.80	4.11 ± 0.84	14.557	0.000
abdominal distension (score)	0.88 ± 0.66	0.91 ± 0.62	0.87 ± 0.74	0.450	0.956

**Table 3:** comparison of AL in three different types of TDT [case (%)]

types	anastomotic leakage	No anastomotic leakage	*P* value
Fr32 silicone tube	1 (1.23)	80 (99.77)	0.301
Fr24 silicone tube	3 (5.56)	51 (94.44)	
Fr32 silicone tube	1 (1.23)	80 (99.77)	0.140
Fr24 latex tubes	3 (6.38)	44 (93.62)	
Fr24 silicone tube Fr24 latex tubes	3 (5.56) 3 (6.38)	51 (94.44) 44 (93.62)	1.000

## Discussion

AL is one of the most severe postoperative complications of rectal cancer. At present, the main factors of AL are considered to be [17, 18]: male, old age, obesity, diabetes, smoking, steroid use, preoperative chemoradiotherapy and intestinal preparation, intraoperative contamination, tumor stage and location, microcirculation disorder, anastomotic tension, operation time and bleeding, etc. Some risk factors of AL can be avoided by strengthening perioperative management, perfecting preoperative preparation, improving the general condition of patients, fine intraoperative operation, protecting the blood supply of anastomosis, and reducing the tension of anastomosis, etc. Despite utilizing these treatments, AL may still occur. Proximal enterostomy can reduce the incidence of anastomotic leakage in high-risk patients. However, it requires the second operation for reimbursement, which not only brings inconvenience to the postoperative life of patients but also increases the pain of the second operation. Many studies have suggested that TDT is effective in reducing AL [8–13, 19–22]. In recent years, it has become a routine practice to retain TDT to prevent AL after the operation. TDT is routinely used in our institution.

Endoluminal pressure is presumed to be associated with AL [23]. TDT can continuously discharge fluid, gas and feces from the proximal large intestine to reduce endoluminal pressure and contamination of the anastomotic site; TDT can also continue to dilate the anus, relieve the closure of the anus, reduce the anastomotic tension, and increase the blood supply of the anastomosis. TDT can also stimulate the anal sphincter and the peripheral nerves in the rectal wall, promoting the recovery of intestinal peristalsis [9]. Therefore, TDT can prevent and reduce rectal AL. However, we find that the size and material of the drainage tube are rarely reported in literature [24–26].

Although our results showed no difference in AL of three different types of anal drainage tubes, the AL of the Fr32 silicone tube was significantly lower than that of the Fr24 latex tubes and the Fr24 silicone tubes (1.23% vs. 5.56% vs. 6.38%). In addition, the time of first drainage and defecation with Fr32 silicone tube after rectal cancer surgery was significantly earlier than that with Fr24 silicone tube and Fr24 latex tube. The results showed that the drainage effect of Fr32 silicone tube was better than that of Fr24 silicone tube and Fr24 latex tube. We think that this may reduce the incidence of AL. The characteristics of the three TDT are analyzed as follows: the Fr32 silicone tube has a larger diameter and is not easy to bend and fold, preventing blood clots and stool from blocking the lumen. In addition, the material of Fr32 silicone tube is flexible, which ensures that it is not prone to distortion and obstruction after compression, so it achieves a good effect of smooth drainage. This is consistent with the results reported in relevant literature that larger TDT is superior to ordinary small diameter drainage tubes [17, 18, 20]. The Fr24 silicone tube has a thin wall and soft texture, and its lumen is easy to fold, become smaller or close, so it cannot achieve the role of adequate drainage. The Fr24 latex tube is mainly made of latex, because of its strong irritability, it is easy to be wrapped by omentum and other tissues and block the lumen in a short time, thus affecting the drainage effect. In clinical practice, It is often used for T-tube drainage of a common bile duct. On the contrary, the silicone tube is almost non-irritating and is not easily encapsulated and blocked by tissue, which is conducive to unobstructed drainage.

The use of TDT also has some common disadvantages, such as anus discomfort symptoms, soreness of the perianal skin, etc. In addition, TDT can make early out-of-bed activities inconvenient for patients, which may increase patients’ bedtime and increase the risk of thrombosis [12], and it even reported that TDT may have association with bowel perforation [12, 22]. Is it possible that TDT is too deep or the diameter of the tube is too large? The compression of the intestinal wall leads to ischemia and necrosis of the intestinal mucosa, leading to intestinal perforation. These aspects deserve further study. However, in our study, Fr32 silicone tube with a diameter of 10 mm was safe and effective, and no case of intestinal perforation caused by TDT was found.

Some limitations of this study should be mentioned. Our study has several limitations. First of all, it is a retrospective study in a consecutive series of selected patients. It’s not a randomized controlled study. Furthermore, we did not evaluate intraluminal pressure in patients with three different types of TDT. Finally, we could not compare the drainage effect of the silicone tube and latex tube under the same size of Fr32 after anterior resection for rectal cancer. These are studies that need further investigation.

## Conclusions

The drainage effect of Fr32 silicone tube is better than that of Fr24 silicone tube and Fr24 latex tube after anterior resection for rectal cancer. Fr32 silicone may better prevent the occurrence of AL, but randomized controlled studies are needed.

## Data Availability

The datasets used during the current study are available from the corresponding author on reasonable request.

## Abbreviations

AL: Anastomotic leakage
TDT: Transanal drainage tubes
CT: Computerized tomography
MRI: Magnetic resonance imaging
